# Nocturnal Hypoxic Exposure Combined with Two-Week Hypoxic Training and Calorie Restriction Improves Lipid Profile and Body Composition in Men with Obesity-Related Hypercholesterolemia: A Controlled Intervention Study

**DOI:** 10.3390/ijms27125151

**Published:** 2026-06-06

**Authors:** Emil Jędrzejewski, Miłosz Czuba, Adam Niemaszyk, Kamila Płoszczyca, Katarzyna Kaczmarczyk, Robert Gajda

**Affiliations:** 1 Department of Surgery, Powiatowe Centrum Zdrowia Sp. z o.o., 05-400 Otwock, Poland; e.jedrzejewski@wp.pl; 2 Faculty of Rehabilitation, Józef Piłsudski University of Physical Education in Warsaw, 00-968 Warsaw, Poland; adam.niemaszyk@awf.edu.pl (A.N.); kamila.ploszczyca@awf.edu.pl (K.P.);; 3 Department of Kinesiology and Health Prevention, Jan Dlugosz University, 42-217 Czestochowa, Poland; gajda@gajdamed.pl

**Keywords:** normobaric hypoxia, nocturnal hypoxic exposure, intermittent hypoxic training, LDL cholesterol, obesity, hypercholesterolemia

## Abstract

Despite advances in lifestyle-based therapy, achieving clinically meaningful reductions in blood lipid levels remains a major challenge in obese men with secondary hypercholesterolemia. Hypoxic exposure encompassing both training sessions and nocturnal rest may offer a novel adjunct to conventional interventions; however, no study has evaluated such a protocol in this population. Twenty sedentary men with obesity-related hypercholesterolemia were randomly allocated to a hypoxic group (H) or normoxic control group (C). Both groups completed an identical two-week high-intensity training program under an individualized calorie-restricted diet, residing at the same lowland location (~100 m above sea level). The H group trained and rested under normobaric hypoxia (FiO_2_ = 14.4%, simulated altitude ~3000 m, 8 h nightly); C remained under normoxic conditions. The H group demonstrated significantly greater reductions in body mass (−4.1%) and fat mass (−11.0%). Significant reductions in total cholesterol (−20.1%), low-density lipoprotein cholesterol (−21.3%), non-high-density lipoprotein cholesterol (−23.1%), atherogenic index of plasma (−42.4%), and Castelli Risk Index I (−19.4%) occurred exclusively in the H group, accompanied by a strong downward trend in Castelli Risk Index II (*p* = 0.072). High-density lipoprotein cholesterol did not change; for triglycerides, a clear downward trend was observed in the H group, approaching statistical significance within-group (*p* = 0.052). The magnitude of cholesterol reduction was significantly associated with body mass and fat loss (*r* = 0.61–0.67). A two-week intervention combining hypoxic training with nocturnal normobaric hypoxic exposure and caloric restriction produces clinically relevant improvements in lipid profile and body composition in men with obesity-related hypercholesterolemia.

## 1. Introduction

The increasing prevalence of obesity, secondary hypercholesterolemia, and metabolic disorders represents one of the foremost challenges in contemporary public health. These conditions are major risk factors for cardiovascular disease [1]. Elevated plasma LDL cholesterol concentration is the primary cause of ischemic heart disease and ischemic stroke in both developed and developing countries [2], and its co-occurrence with obesity substantially worsens cardiovascular prognosis and limits the effectiveness of non-pharmacological interventions.

The conventional non-pharmacological approach to the management of obesity and hypercholesterolemia relies primarily on lifestyle modification, encompassing dietary caloric restriction and regular physical activity. However, despite the widespread application of calorie-restricted diets and exercise, improvements in lipid profile are frequently limited and transient [3]. These limitations have prompted researchers to explore complementary therapeutic strategies capable of amplifying the effects of conventional dietary and exercise-based interventions.

In this context, growing interest has focused on controlled hypoxic exposure as an adjunct to standard therapeutic management, with the aim of supporting body mass reduction and favorably modifying lipid metabolism. Under controlled conditions, hypoxia activates compensatory mechanisms leading to increased blood oxygen-carrying capacity, enhanced fat oxidation, improved glucose metabolism, and greater insulin sensitivity [4]. These mechanisms are relatively well characterized at the molecular level. Hypoxia activates the transcription factor HIF-1α (hypoxia-inducible factor 1 alpha), which regulates the expression of genes involved in cholesterol transport, fatty acid oxidation, and endothelial function [5]. The beneficial effect of hypoxia on blood lipid concentrations may also be attributed to an increased rate of lipid oxidation resulting from elevated expression of mRNA encoding PGC-1α (peroxisome proliferator-activated receptor gamma coactivator 1-alpha), a protein that induces mitochondrial biogenesis and plays a key role in regulating fatty acid oxidation in skeletal muscle [6]. Consequently, hypoxia may reduce LDL-C concentration through the concerted action of several mechanisms. Activation of HIF-1α and upregulation of PGC-1α enhance fatty acid oxidation and lipid utilization as an energy substrate [5,6], while increased expression of LDL receptors in hepatocytes accelerates lipoprotein catabolism and cholesterol clearance from the circulation [7]. These adaptations are of particular relevance in the context of obesity, in which lipid metabolism is frequently impaired due to insulin resistance, chronic low-grade inflammation, and hepatic steatosis [8].

To date, therapeutic strategies employing hypoxia have focused primarily on intermittent hypoxic training (IHT)—a method combining physical exercise with controlled exposure to a reduced-oxygen environment (FiO_2_ 13–16%), simulating moderate-altitude conditions [9]. In our previous study [10], we demonstrated that a four-week IHT-based therapeutic protocol can effectively improve lipid profile and body composition in men with obesity and secondary hypercholesterolemia, but only when combined with a controlled calorie-restricted diet providing a negative energy balance. A review of the available literature indicates that most IHT-based interventions carried out to date did not incorporate a controlled diet with a negative energy balance, and in many cases provided no dietary supervision whatsoever [11,12,13]. In light of the pathophysiology of obesity and hypercholesterolemia, the absence of dietary control represents a significant methodological limitation: individuals with overweight or obesity typically maintain a positive energy balance, and physical activity—even when augmented by hypoxia—may compensatorily increase appetite and energy intake [14], thereby offsetting the potential metabolic benefits of the intervention. In our previous study [10], a significant correlation was identified between body mass reduction and improvement in total cholesterol concentration (*r* = 0.45; *p* < 0.05), clearly indicating that a negative energy balance is an essential prerequisite for the effectiveness of hypoxic interventions in the treatment of obesity and secondary hypercholesterolemia.

This raises the question of whether shortening the intervention period while simultaneously increasing the hypoxic stimulus dose—by adding nocturnal hypoxic exposure—could yield comparable metabolic effects. This line of reasoning is further supported by our earlier findings concerning changes in lipid profile following nocturnal exposure to normobaric hypoxia [15]. It should be noted that nocturnal exposure to controlled normobaric hypoxia as a distinct therapeutic strategy remains poorly investigated—the limited available evidence pertains mainly to the effects of sleep at high altitude or passive hypobaric hypoxic exposure, rather than to a controlled normobaric model combining IHT with nocturnal hypoxia [16]. This represents a significant gap in the current state of knowledge, which the present study aims to address. Collectively, these observations indicate that the duration of hypoxic exposure may constitute a critical determinant of the effectiveness of the hypoxic stimulus in modifying lipid profile.

To date, no study has simultaneously evaluated the effects of IHT combined with nocturnal normobaric hypoxic exposure and a controlled hypocaloric diet on lipid profile and body composition in individuals with obesity and secondary hypercholesterolemia. We hypothesized that a two-week intensive hypoxic training program under normobaric conditions (IHT), combined with nocturnal hypoxic exposure and a controlled diet providing a negative energy balance, would lead to favorable changes in lipid profile, atherogenic indices, and body composition. Furthermore, we anticipated that increasing the daily hypoxic dose by combining IHT with nocturnal exposure would yield comparable or greater lipid-related adaptations than those observed with hypoxic training alone.

The aim of the present study was therefore to determine changes in blood lipid profile markers and body composition in obese men with secondary hypercholesterolemia, without coexisting medical conditions, in response to a two-week intensive training program conducted under normobaric hypoxia combined with nocturnal hypoxic exposure and a controlled hypocaloric diet.

## 2. Results

### 2.1. Changes in Body Mass and Body Composition

ANOVA with repeated measures for the group × training interaction revealed statistically significant differences in body mass (BM; *F* = 27.49, *p* < 0.001), body fat percentage (%FAT; *F* = 4.59, *p* < 0.05), and fat mass (FM; *F* = 10.51, *p* < 0.05). No significant changes in resting metabolic rate (RMR) were detected in either group based on indirect calorimetry assessments (Table 1).

**Figure 1:**
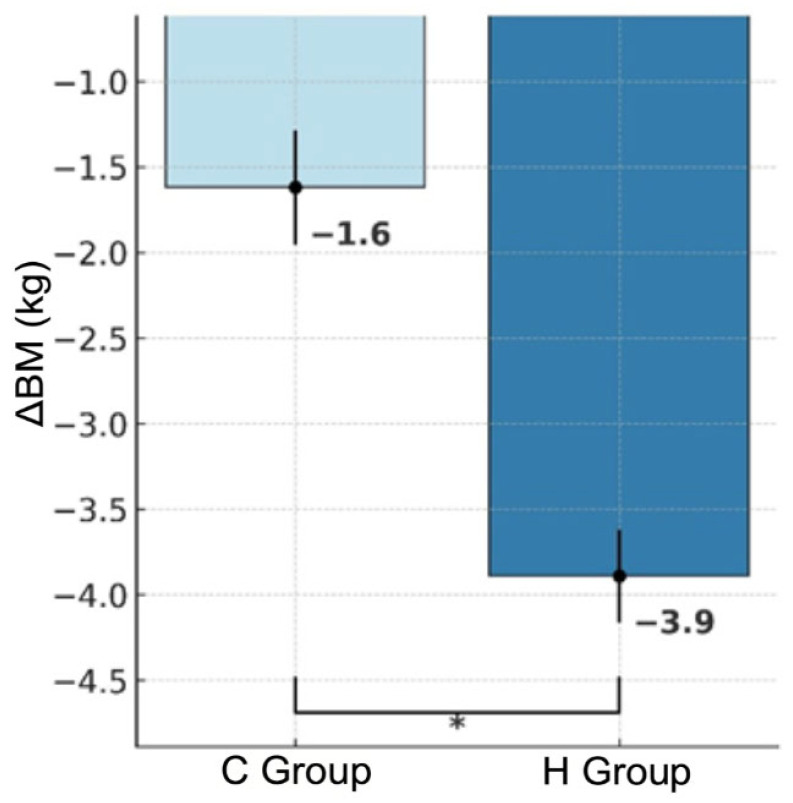
Post-intervention changes in body mass in hypoxic and control groups; * *p* < 0.05, significant differences between groups. Abbreviations: ΔBM—change in body mass, H group—hypoxic training group, C group—control group.

Tukey’s post hoc analysis indicated a significant reduction in BM in both groups, amounting to 4.1% (*p* < 0.001; *g* = 0.48 (95% CI: −0.38–1.33)) in the H group and 1.8% (*p* < 0.001; *g* = 0.18 (95% CI: −1.06–0.70)) in the C group (Table 1). However, independent-samples *t*-test analysis of BM delta values (∆BM) demonstrated a significantly greater reduction in the H group, which was exposed to hypoxia both during nocturnal rest and training sessions, compared with the C group (*t* = 5.24, *p* < 0.001; *g* = 2.34 (95% CI: 1.10–3.58); Figure 1).

**Figure 2:**
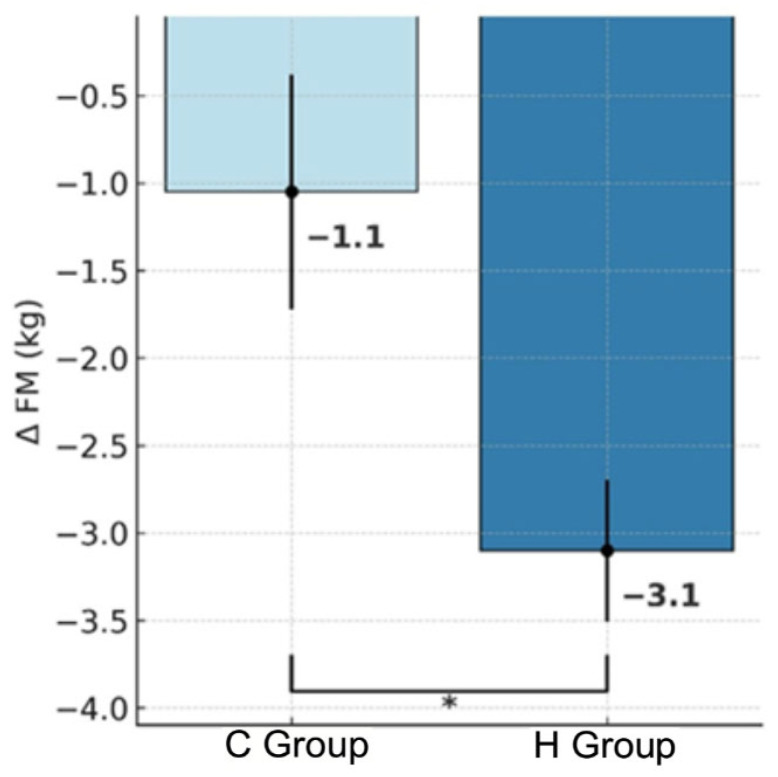
Post-intervention changes in fat mass in hypoxic and control groups; * *p* < 0.05, significant differences between groups. Abbreviations: ΔFM—change in fat mass, H group—hypoxic training group, C group—control group.

**Figure 3:**
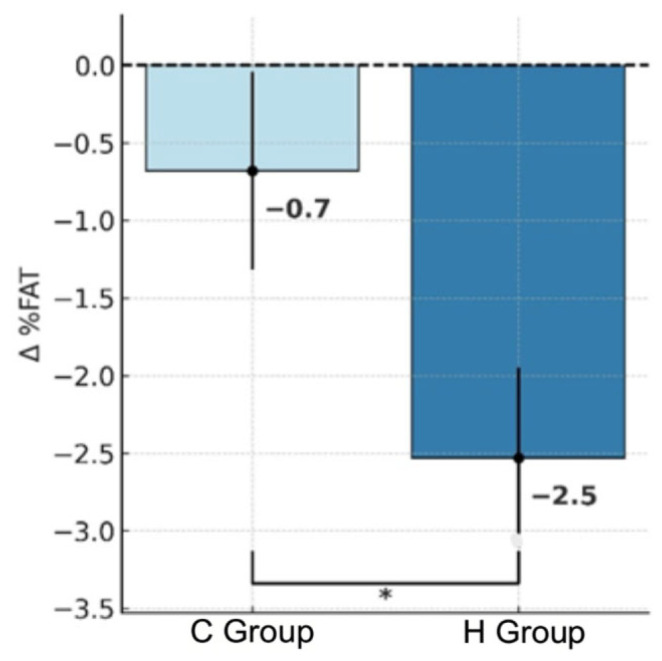
Post-intervention changes in percentage of body fat in hypoxic and control groups; * *p <* 0.05, significant differences between groups. Abbreviations: Δ%FAT—change in percentage of body fat, H group—hypoxic training group, C group—control group.

In the H group, hypoxic exposure was also accompanied by a significant reduction in FM of 11.0% (*p* < 0.001; *g* = 0.52 (95% CI: −0.34–1.37), along with a significant decrease in %FAT of 8.8% (*p* < 0.05; *g* = 0.44 (95% CI: −0.41–1.29). In contrast, no statistically significant changes in FM or %FAT were observed in the C group (Table 1). Between-group comparisons of ∆FM and ∆%FAT confirmed that the changes recorded in the H group were statistically significant relative to the C group (*p* < 0.05; ∆FM: *g* = 1.12 (95% CI: 0.13–2.11); ∆%FAT: *g* = 0.92 (95% CI: −0.03–1.87); Figure 2 and Figure 3).

### 2.2. Changes in Lipid Profile

**Figure 4:**
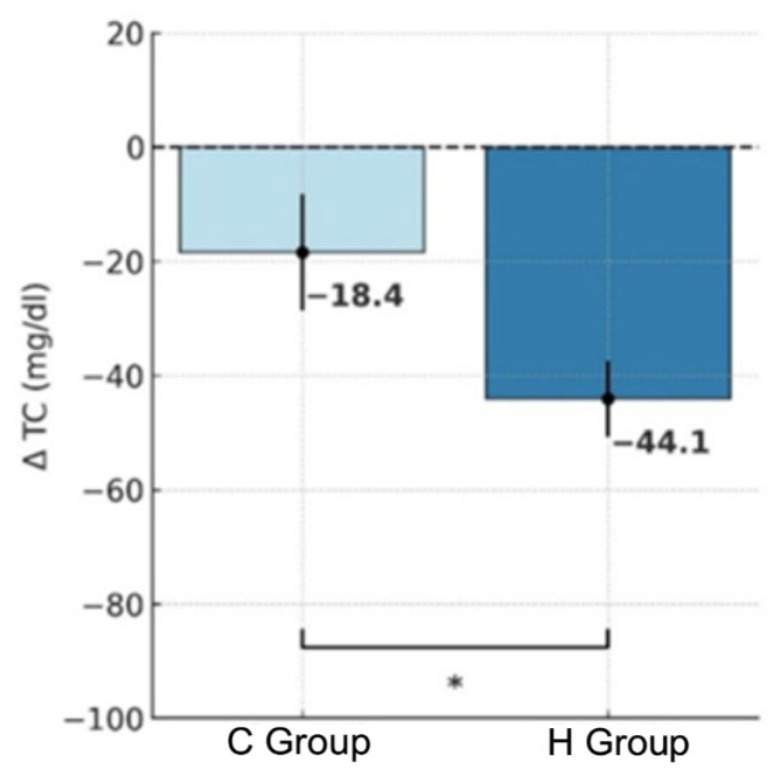
Post-intervention changes in total cholesterol concentrations in hypoxic and control groups; * *p* < 0.05, significant differences between groups. Abbreviations: ΔTC—change in total cholesterol concentrations, H group—hypoxic training group, C group—control group.

**Figure 5:**
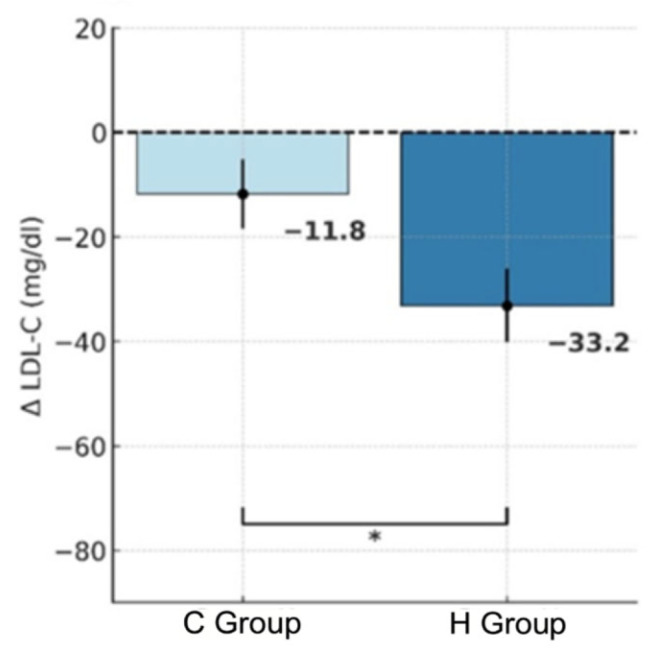
Post-intervention changes in LDL cholesterol concentrations in hypoxic and control groups; * *p* < 0.05, significant differences between groups. Abbreviations: ΔLDL-C—change in low-density lipoprotein cholesterol, H group—hypoxic training group, C group—control group.

**Figure 6:**
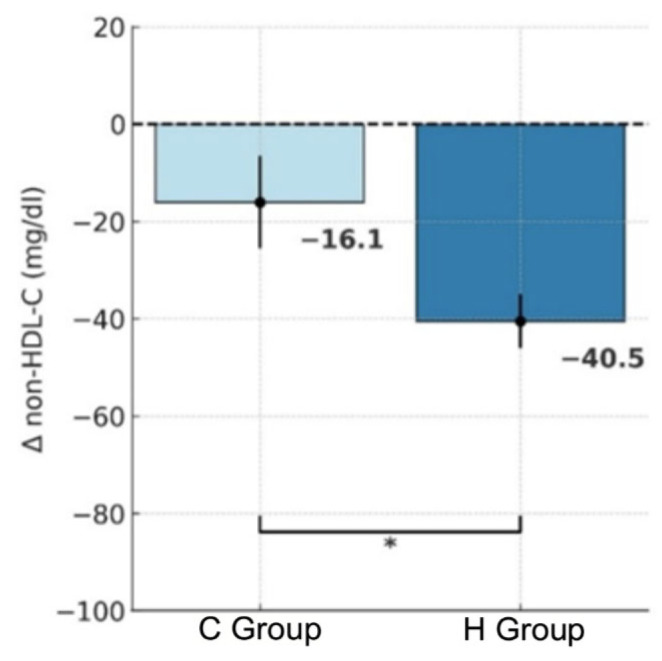
Post-intervention changes in non-HDL cholesterol concentrations in hypoxic and control groups; * *p* < 0.05, significant differences between groups. Abbreviations: Δnon-HDL-C—change in non-high-density lipoprotein cholesterol, H group—hypoxic training group, C group—control group.

Repeated measures ANOVA revealed a significant effect of hypoxic conditions on total cholesterol (TC; *F* = 4.45, *p* < 0.05), low-density lipoprotein cholesterol (LDL-C; *F* = 4.91, *p* < 0.05), and non-high-density lipoprotein cholesterol (non-HDL-C; *F* = 4.92, *p* < 0.05). Tukey’s post hoc analysis indicated a significant reduction in TC by 20.1% following the two-week intervention in the H group (*p* < 0.001; *g* = 1.59 (95% CI: 0.60–2.58); Table 2). The *t*-test further confirmed that this decrease was significantly greater in the H group compared with the C group (*p* < 0.05, *g* = 0.91 (95% CI: −0.03–1.86); Figure 4). A similar pattern of change was observed for LDL-C and non-HDL-C following the intervention. In the H group, LDL-C concentration decreased significantly by 21.3% (*p* < 0.01; *g* = 1.47 (95% CI: 0.50–2.43)), while non-HDL-C fell markedly by 23.1% (*p* < 0.001; *g* = 1.73 (95% CI: 0.72–2.75); Table 2). Between-group comparisons revealed significantly greater ∆LDL-C and ∆non-HDL-C in the H group relative to the C group (*p* < 0.05; ∆LDL-C: *g* = 0.97 (95% CI: 0.01–1.93); ∆non-HDL-C: *g* = 0.95 (95% CI: −0.01–1.91); Figure 5 and Figure 6). No comparable changes in TC, LDL-C, or non-HDL-C were observed in the C group, which trained and resided under normoxic conditions throughout the two-week intervention period. HDL-C concentrations did not change significantly in either group (Table 2). For TG, the group × time interaction was not significant (*p* > 0.05); however, within-group comparison in the H group revealed a borderline reduction (*p* = 0.052; *g* = 0.40 (95% CI: −1.29–0.48)), while no change was observed in C (*p* = 0.92). Moreover, a between-group comparison of percentage changes in TG (H: −29.0% vs. C: +8.7%) using the Mann–Whitney U test also approached the predefined significance threshold (*p* = 0.053; *g* = 0.64 (95% CI: −0.27–1.55)), suggesting a possible hypoxia-related reduction in TG concentrations that nevertheless did not reach statistical significance in the present sample. The relatively large standard deviations observed for TG concentrations in both groups indicate substantial inter-individual variability, which, together with the small sample size, may have reduced the precision of the statistical estimates and contributed to the relatively wide confidence intervals.

**Figure 7:**
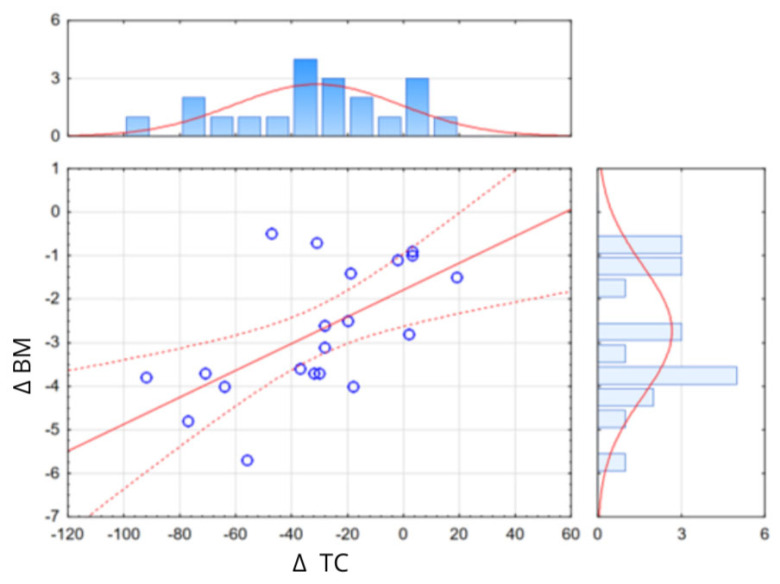
Correlation between delta values for body mass (ΔBM) and delta values of total cholesterol (∆TC) after the experiment in both groups. Abbreviations: ΔBM—change in body mass, ΔTC—change in total cholesterol; solid line—regression line; dot line—95% confidence interval; circle—individual data points for each participant.

**Figure 8:**
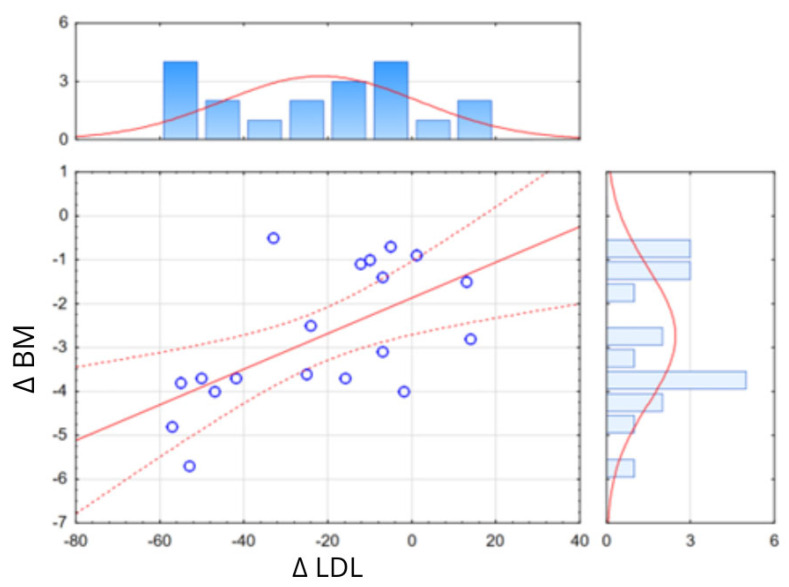
Correlation between delta values for body mass (ΔBM) and delta values of LDL cholesterol (ΔLDL-C) after the experiment in both groups. Abbreviations: ΔBM—change in body mass, ΔLDL-C—change in LDL cholesterol concentration; solid line—regression line; dot line—95% confidence interval; circle—individual data points for each participant.

**Figure 9:**
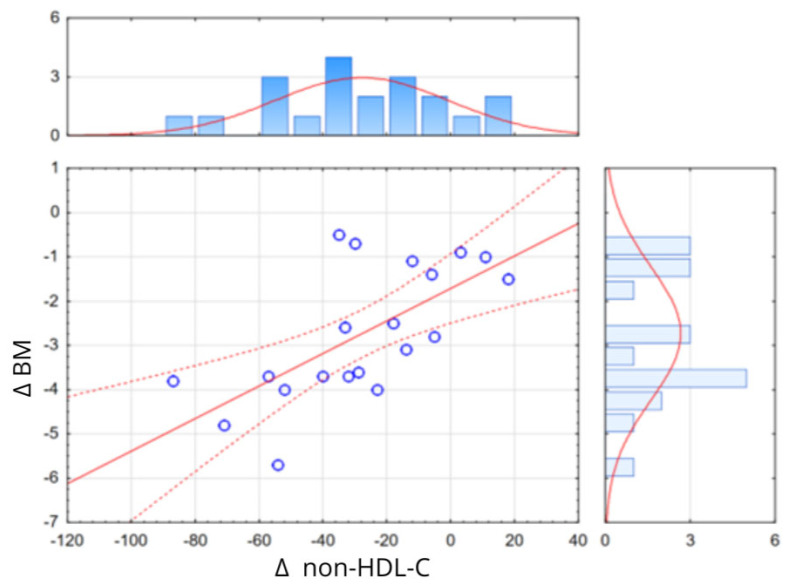
Correlation between delta values for body mass (ΔBM) and delta values of non-HDL cholesterol (Δnon-HDL-C) after the experiment in both groups. Abbreviations: ΔBM—change in body mass, Δnon-HDL-C—change in non-HDL cholesterol concentration; solid line—regression line; dot line—95% confidence interval; circle—individual data points for each participant.

**Figure 10:**
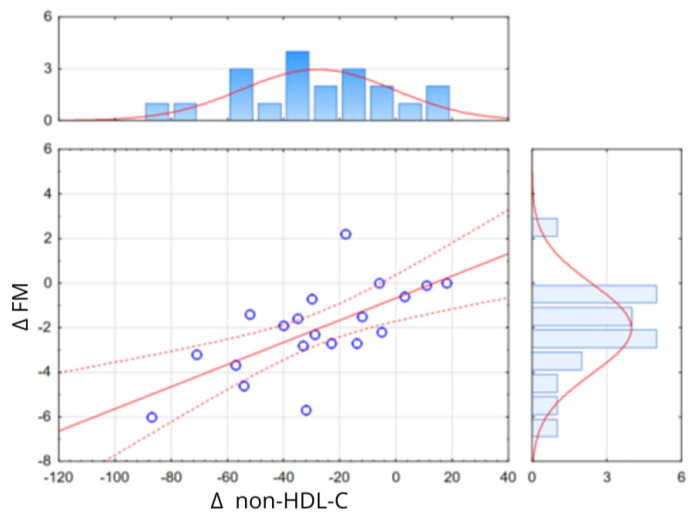
Correlation between delta values for fat mass (ΔFM) and delta values of non-HDL cholesterol (Δnon-HDL-C) after the experiment in both groups. Abbreviations: ΔFM—change in fat mass, Δnon-HDL-C—change in non-HDL cholesterol concentration; solid line—regression line; dot line—95% confidence interval; circle—individual data points for each participant.

Correlation analysis revealed significant associations between changes in anthropometric parameters and improvements in lipid profile. Body mass reduction (∆BM) was significantly correlated with the decrease in total cholesterol concentration (∆TC; *r* = 0.61, *p* < 0.05, Figure 7), indicating that participants who experienced greater body mass loss also demonstrated more pronounced improvements in lipid parameters. A similar relationship was identified between ∆BM and the reduction in LDL-C (*r* = 0.61, *p* < 0.05; Figure 8) and non-HDL-C (*r* = 0.66, *p* < 0.05; Figure 9). These findings suggest that even a short-term hypoxic intervention leading to body mass reduction contributes to favorable changes in the lipid fractions of greatest diagnostic relevance for cardiovascular risk assessment. Notably, a strong correlation was also found between the reduction in fat mass (∆FM) and the decrease in non-HDL-C concentration (∆non-HDL-C; *r* = 0.67, *p* < 0.05; Figure 10), further highlighting the central role of fat mass reduction in improving lipid profile. Collectively, these results confirm that anthropometric changes—particularly the reduction in fat mass—represent a key factor modulating the metabolic response to hypoxic training interventions.

All three atherogenic indices (Atherogenic index of plasma, AIP; Castelli’s risk index I, CRI-I; Castelli’s risk index II, CRI-II) decreased in the H group, with reductions of 42.4%, 19.4%, and 20.5%, respectively, while values in the C group remained essentially unchanged (Table 3). The Wilcoxon test confirmed a significant 42.4% reduction in AIP in the H group (Z = 2.701, *p* < 0.01; *g* = 0.66 (95% CI: −0.21–1.53)), and a paired *t*-test demonstrated a significant 19.4% reduction in CRI-I (*t* = 2.90, *p* < 0.05; *g* = 0.85 (95% CI: −0.03–1.74), Table 3). For CRI-II, a clear downward trend was observed in the H group (−20.5%), approaching the predefined significance threshold (*t* = 2.06, *p* = 0.072; *g* = 0.66 (95% CI: −1.56–0.24)), whereas no change was noted in C. Importantly, none of the analyzed atherogenic indices changed significantly in the C group, indicating that the observed improvements were specific to the hypoxic condition.

## 3. Discussion

The present study provides the first controlled evidence that a two-week normobaric hypoxic intervention combining IHT with passive nocturnal hypoxic exposure, conducted alongside a controlled hypocaloric diet, produces significantly greater improvements in lipid profile and body composition than an otherwise identical intervention performed under normoxic conditions in men with overweight or obesity and secondary hypercholesterolemia. Specifically, the H group showed significantly greater reductions in BM and FM than the C group, and a significant reduction in %FAT was observed exclusively in the H group. The hypoxic intervention was further associated with significant reductions in TC, LDL-C, and non-HDL-C, none of which reached significance in the C group. TG and HDL-C concentrations did not change significantly in either group. A significant improvement in the atherogenic index of plasma (AIP) was recorded exclusively in the H group. The magnitude of lipid improvement was proportional to the magnitude of weight and fat loss: ∆BM correlated significantly with ∆TC, ∆LDL-C, and ∆non-HDL-C, while ∆FM correlated with ∆non-HDL-C.

### 3.1. Caloric Deficit as a Prerequisite for the Effectiveness of IHT

The changes demonstrated in the present study—reductions in BM, FM, and atherogenic lipid fractions observed following hypoxic exposure—are consistent with a growing body of evidence supporting the therapeutic potential of IHT in individuals with metabolic disorders. These findings directly extend our earlier observations [10], in which a four-week IHT protocol combined with a controlled hypocaloric diet produced significant reductions in BM (−5.4%), FM (−14.7%), TC (−22.6%), LDL-C (−25.8%), and non-HDL-C (−26.5%) in a comparable population of men with obesity and secondary hypercholesterolemia. Additionally, a recent meta-analysis by He et al. [17] demonstrated that IHT reduces FM and improves BMI more effectively than normoxic training in middle-aged and older adults. Similarly, Ramos-Campo et al. [18] reported a stronger effect of hypoxic versus normoxic training on triglyceride reduction and body composition.

The findings of the present study extend these observations to a clinically defined population with confirmed secondary hypercholesterolemia. Importantly, the absence of significant changes in the C group—despite adherence to an identical training schedule and dietary protocol—suggests that hypoxia, rather than physical activity or caloric restriction alone, is the key determinant of the superior metabolic outcomes observed in the H group. This interpretation is further supported by the significant between-group differences in TC, LDL-C, non-HDL-C, and AIP, none of which were observed in participants training under normoxic conditions.

At the same time, the present findings corroborate the conclusion drawn in our previous study [10] that a controlled hypocaloric diet providing a negative energy balance is an essential prerequisite for achieving clinically meaningful effects of IHT on lipid metabolism in individuals with obesity. Earlier studies employing IHT without dietary control consistently failed to demonstrate significant improvements in lipid profile or body composition [11,12,13]. This may be explained by the fact that individuals with excess adipose tissue typically maintain a positive energy balance, and physical activity—even when augmented by hypoxia—may compensatorily stimulate appetite and increase caloric intake [14], thereby neutralizing the metabolic stimulus. The significant correlations between ∆BM and ∆TC (*r* = 0.61; *p* < 0.05) and between ∆FM and ∆non-HDL-C (*r* = 0.67; *p* < 0.05) observed in the present study confirm that reductions in BM and FM remain essential mediators of lipid improvement, regardless of the environmental conditions under which training is conducted.

### 3.2. Daily Hypoxic Dose as a Key Determinant of Lipid Adaptation

A novel finding of the present study is that the addition of a daily passive hypoxic stimulus during nocturnal rest significantly enhances the metabolic response to IHT combined with a caloric restriction diet.

The rationale for this approach derives from our earlier research. In Płoszczyca et al. [15], a three-week LH-TL protocol involving 11–12 h of daily normobaric hypoxic exposure (FiO_2_ = 16.5%, simulated altitude~2000 m a.s.l.) produced significant improvements in all analyzed lipid parameters in trained cyclists, despite a normal baseline lipid profile and the absence of a caloric restriction diet. In the LH-TL group, statistically significant changes were observed across all analyzed lipid profile parameters: HDL-C increased by 9.0%, while TC decreased by 9.2%, LDL-C by 18.2%, and TG by as much as 27.6%. Concurrent significant improvements were observed in atherogenic indices—AIP, CRI-I, and CRI-II—indicating a favorable reduction in cardiovascular risk. No significant changes in lipid profile or atherogenic indices were found in the IHT or control groups, suggesting that exercise training under normoxic conditions alone was insufficient to induce these adaptations. The absence of a response in the IHT group—which appears to conflict with the findings of our previous study [10]—was most likely attributable to the normal baseline lipid profile of the participants and the absence of a negative energy balance. This is consistent with earlier IHT studies in which no controlled caloric restriction diet was implemented [11,12,13]. It is worth emphasizing, however, that the favorable changes in lipid profile observed in the LH-TL group were achieved despite a normal baseline lipid profile and the absence of a caloric restriction diet. This suggests that a sufficiently strong and prolonged hypoxic stimulus—in the form of multi-hour daily exposure—is capable of independently inducing adaptations in lipid metabolism. This discrepancy between protocols differing primarily in the duration of daily hypoxic exposure provided the conceptual basis for the present study and suggested that the total daily hypoxic dose, rather than hypoxic training alone, may be the key determinant of lipid adaptation.

Indirect support for the strategy of increasing the daily hypoxic dose comes from epidemiological data on populations residing at high altitude, though these should be treated as contextual background rather than a direct equivalent of a short-term intervention. Individuals permanently residing in high-mountain regions exhibit a reduced risk of CVD and lower all-cause mortality, which has been linked to a favorable lipid profile—lower TC and LDL-C values alongside elevated HDL-C concentrations compared with lowland inhabitants [19,20,21,22]. Favorable lipid changes have also been documented in participants of high-altitude expeditions [23,24] and in individuals spending time at moderate altitudes [25,26], where even a stay of several days was found to be associated with reductions in TC of 6–13% and LDL-C of 10–14% [26,27]. Regarding HDL-C, findings remain inconsistent [26]. An analogous pattern—an inverse relationship between altitude of residence and obesity prevalence or BMI—has been demonstrated in American [28], Peruvian [29], and Tibetan [30] populations. It should be emphasized, however, that these data reflect the effects of chronic, often multigenerational adaptation to hypoxia and cannot be directly extrapolated to a two-week intervention in lowland individuals.

The findings of the present study regarding TC and LDL-C are consistent with these observations. At the same time, no significant changes in HDL-C concentration were observed in either group, and TG concentrations did not change significantly, despite a numerical downward trend in the H group. The absence of HDL-C improvement is consistent with the findings of Jędrzejewski et al. [10] and Gao et al. [31], and most likely reflects the magnitude of the caloric deficit applied—rapid body mass reduction under hypocaloric diet conditions suppresses the rise in HDL-C and may even reduce its circulating concentration [32]. Restricting dietary fat intake to approximately 20% of total energy—implemented to achieve an anticatabolic effect through increased protein contribution—may have additionally attenuated the HDL-C response [33]. The absence of a significant TG reduction, in contrast to the four-week protocol [10], likely reflects the shorter duration of the intervention and the high interindividual variability in baseline TG concentrations.

From a molecular mechanistic perspective, prolonged daily hypoxic exposure should sustain HIF-1α activation beyond the peri-exercise window examined in our previous study [10], maintaining upregulation of genes involved in cholesterol transport, fatty acid oxidation, and hepatocytes, across an extended portion of the day [5,7]. Concurrently, prolonged hypoxic exposure may favor the maintenance of elevated PGC-1α expression, supporting mitochondrial biogenesis and enhanced lipid oxidation both during nocturnal rest and subsequent training sessions [6]. It should be emphasized, however, that the mechanisms outlined above remain speculative, as the present study did not directly assess gene expression or molecular markers related to HIF-1α or PGC-1α activation.

### 3.3. Comparison with Our Previous Four-Week IHT Protocol [10]

Indirect evidence suggesting the potential additional therapeutic value of nocturnal hypoxic exposure is provided by a comparison of the present study with our earlier protocol based on the same methodology and dietary approach [10]. Both studies enrolled physically inactive men with overweight or obesity and secondary hypercholesterolemia, employed an identical high-intensity training protocol and identical nutritional protocol, and were conducted in the same controlled residential environment. The only differences were the duration of the intervention (2 weeks vs. 4 weeks) and the addition of nocturnal hypoxic exposure in the present protocol.

Despite an intervention period half as long, the present study yielded improvements in lipid profile comparable to those reported by Jędrzejewski et al. [10], and in several respects more pronounced. In the previous study [10], the four-week IHT protocol produced significant reductions in TC of 22.6%, LDL-C of 25.8%, and non-HDL-C of 26.5%, alongside a significant improvement in AIP of 24.4%; no significant changes were observed in the remaining atherogenic indices (CRI-I, CRI-II). In the present two-week intervention, TC decreased significantly by 20.1%, LDL-C by 21.3%, and non-HDL-C by 23.1%, while AIP improved significantly by 42.4% and CRI-I by 19.4%; a clear downward trend was additionally observed for CRI-II. The magnitude of AIP improvement in the present study was therefore nearly twice that reported in the previous study. Notably, CRI-I also improved significantly in the present protocol, indicating a broader range of favorable atherogenic adaptations. These findings are of particular clinical relevance given that AIP is considered one of the most sensitive composite markers of atherogenic risk, reflecting the balance between pro- and antiatherogenic lipoprotein fractions [34], and CRI-I—a classical index of coronary heart disease risk [35,36]—is an established predictor of cardiovascular events [37]. Taken together, these observations suggest that the applied procedure may translate into a reduction in the estimated cardiovascular risk of the participants. It should be noted, however, that this comparison is indirect in nature and does not substitute for a direct randomized study comparing both protocols in the same population. In statin meta-analyses, each 1 mmol/L reduction in LDL-C is associated with an approximately one-fifth (~20–22%) lower risk of major vascular events [38]. The reduction observed here (≈0.96 mmol/L) is of a similar magnitude, although—as cardiovascular events were not assessed—any benefit on clinical outcomes remains speculative and would need to be confirmed in dedicated long-term studies.

It should be noted that a reduction in LDL-cholesterol does not invariably indicate a cardioprotective effect. Such changes may also be observed in the context of chronic inflammation, malnutrition, or hepatic dysfunction. Consequently, low LDL-cholesterol may in certain cases reflect adverse biological processes rather than a favorable metabolic adaptation—a phenomenon referred to as the “cholesterol paradox” [39,40]. This phenomenon has been documented in acute coronary syndrome, where low admission LDL was associated with worse prognosis—mediated by chronic inflammation and frailty [40]—as well as in heart failure, where lower LDL levels paradoxically correlate with increased mortality [41]. In the present study, however, participants were otherwise healthy individuals with no chronic disease and no clinical signs of malnutrition or systemic illness at enrolment. Importantly, inflammatory blood count parameters, including white blood cell count and differential, showed no significant changes throughout the intervention, providing further evidence against the presence of inflammation as an alternative explanation for the observed LDL reduction. Although dedicated inflammatory markers were not assessed, which represents a limitation of the present study, inflammation as the underlying cause of the observed LDL reduction appears unlikely.

Furthermore, the associations between anthropometric and lipid changes were stronger in the present study. The correlation between ∆BM and ∆TC was r = 0.61 in the present study, compared with r = 0.45 in Jędrzejewski et al. [10], suggesting that nocturnal hypoxic exposure not only accelerates lipid-lowering but also strengthens the relationship between fat loss and cholesterol reduction. A similar pattern was observed for ∆BM and ∆LDL-C (*r* = 0.61) and ∆FM and ∆non-HDL-C (*r* = 0.67). Taken together, these observations indicate that multi-hour hypoxic exposure acts as an additional metabolic stimulus, amplifying the effects of both caloric restriction and IHT on lipid metabolism. It should be noted that reductions in BM and FM in the H group of the present study (−4.1% and −11.0%, respectively) were marginally smaller than those reported in the previous four-week protocol (−5.4% and −14.7%), which is attributable to the longer duration of that intervention and the correspondingly greater cumulative caloric deficit achieved.

The observation regarding TG concentrations warrants separate commentary. Despite a clear downward trend in the H group, TG did not reach formal statistical significance. Repeated measures ANOVA revealed no significant group × time interaction for TG, although within-group analysis indicated a borderline reduction in the H group (*p* = 0.052), with no change observed in the C group (*p* = 0.92). Furthermore, between-group comparison of percentage changes in TG (H: −29.0% vs. C: +8.7%) also revealed a difference approaching the predefined significance threshold (*p* = 0.053), further supporting the presence of a consistent, albeit formally non-significant, hypoxia-related TG-lowering effect. The direction of change was consistent across 9 of 10 participants in the H group (−35%) and is in agreement with the significant improvement in AIP (−42.4%), which reflects a reduction in the TG/HDL-C ratio with HDL-C remaining unchanged. The observed pattern therefore suggests a genuine biological effect whose detection at the between-group level was limited by the small sample size and the marked heterogeneity of responses in the C group (in which 6 of 10 participants showed an increase in TG).

This discrepancy between the significant reduction in TC and LDL-C and the merely trend-level decrease in TG—also in contrast to our four-week protocol [10]—may be explained by the differing regulatory timeframes of individual lipid fractions. TC and LDL-C are governed by hepatic cholesterol synthesis and LDL receptor expression via the SCAP/SREBP-2 axis [7], and caloric deficit rapidly modifies these pathways in the liver, leading to normalization of cholesterol synthesis within the first days of a dietary intervention [42]. TG reduction, by contrast, depends largely on lipoprotein lipase (LPL) activity in skeletal muscle, which is upregulated by physical training [43]. These changes co-occur with adaptations that increase muscle oxidative capacity, such as mitochondrial biogenesis and enhanced fatty acid oxidation [44]. A two-week intervention may therefore have been insufficient for the full development of this muscular adaptation, which explains the observed discrepancy between the rapid cholesterol response and the delayed, merely trending TG response. The consistency of the direction of TG changes with the significant improvement in AIP and with the reductions in TC, LDL-C, and non-HDL-C nevertheless indicates the internal coherence of the observed metabolic adaptation profile.

The heterogeneous pattern of TG changes observed in the C group warrants additional consideration. In this group, TG concentrations increased in 6 of 10 participants despite a maintained caloric deficit and regular training. In individuals with central obesity, enhanced free fatty acid (FFA) flux to the liver leads to increased re-esterification into TG and overproduction of VLDL particles, while the accompanying reduction in skeletal muscle LPL activity further limits TG lipolysis [8,45]. Under conditions of a short-term dietary and exercise intervention, these mechanisms may transiently dominate over TG-lowering processes, particularly under normoxia, where—in contrast to hypoxia—there is no additional stimulation of skeletal muscle LPL activity or fatty acid β-oxidation. Hypoxic exposure may, however, restore the balance between fat mobilization and utilization through the concurrent activation of HIF-1α- [5], AMPK- [46], and PGC-1α-dependent pathways [6], which increase skeletal muscle oxidative capacity [6,47] and may improve insulin sensitivity [48].

### 3.4. Clinical Implications and Limitations of the Study

From a clinical standpoint, the findings of the present study carry significant practical implications. The two-week residential protocol combining IHT with nocturnal hypoxic exposure and a supervised hypocaloric diet produced clinically meaningful improvements in the most diagnostically relevant lipid fractions—LDL-C, non-HDL-C, and AIP—within a timeframe compatible with short-term therapeutic hospitalization or a rehabilitation program. The absence of significant changes in FFM and RMR in either group confirms that the dietary strategy effectively preserved muscle mass, which is an important consideration in the context of obesity treatment.

These findings support the concept that increasing the daily hypoxic dose through nocturnal exposure represents a time-efficient strategy for accelerating metabolic adaptation in individuals with obesity-related hypercholesterolemia. This has direct implications for the design of future hypoxia-based therapeutic programs, suggesting that hypoxic exposure duration should be treated as a primary dosing variable when planning interventions.

Several limitations of the present study should be acknowledged. The first is the relatively small sample size (*n* = 20), which limits the generalizability of the findings to a broader population. It should be noted, however, that despite the small sample, a controlled hypocaloric diet, a standardized and supervised training program, and supervised nocturnal hypoxic exposure in hotel rooms were all implemented, which enhanced measurement reliability and reduced the influence of confounding factors. Several outcomes (%FAT, FM, and BM) showed small-to-moderate effect sizes; however, the corresponding confidence intervals were relatively wide and often included zero, reflecting the limited precision of the estimates due to the small sample size. Therefore, these findings should be interpreted with caution. In contrast, lipid-related variables (TC, LDL-C, and non-HDL-C) demonstrated large effect sizes with confidence intervals that remained above zero, providing stronger evidence for clinically meaningful intervention-induced improvements in the lipid profile.

The second limitation is the relatively short intervention duration (2 weeks), which did not permit verification of the durability of the observed changes over a longer time horizon. The present study is, however, part of a broader project aimed at developing and implementing a training protocol capable of effectively modifying lipid profile, body composition, and cardiovascular indices in individuals with obesity-related hypercholesterolemia. Given the requirement for participants to remain in controlled conditions—necessitated by the nature of hypoxic training and dietary supervision—the protocol duration was deliberately shortened to ensure practical feasibility and potential applicability in real-world settings. In our view, the durability of the observed effects will depend primarily on the extent to which participants consolidate changes in dietary habits and lifestyle, as these are essential for sustaining body mass reduction and favorable changes in lipid profile.

Further limitations include the restriction of the study population to men aged 20–40 years. Women were deliberately excluded to eliminate the influence of hormonal fluctuations associated with the menstrual cycle on lipid metabolism parameters. This approach enhanced the internal validity of the study design while limiting the transferability of findings to women and older individuals. Additionally, the study design did not include a diet-only control group, as the aim was to isolate the specific contribution of hypoxia within an identical combined diet-and-exercise protocol. The absence of significant changes in lipid profile in the normoxic group (C) confirms that a hypocaloric diet alone—under the conditions applied—was insufficient to produce the observed improvements in lipid parameters. Maintaining identical nutritional and training protocols across both groups allowed the specific effect of hypoxia on lipid profile and body composition to be isolated. A further limitation is that inflammatory markers (e.g., CRP, IL-6, IL-1, TNF-α, IL-10) were not assessed; given the combined stress of caloric restriction, exercise, and hypoxic exposure, evaluating the inflammatory response is an important objective for future studies.

## 4. Materials and Methods

### 4.1. Participants

The study involved 20 physically inactive men aged 20–40 years with overweight or obesity and secondary hypercholesterolemia consistent with an unhealthy lifestyle (i.e., physical inactivity and poor dietary habits), without coexisting medical conditions. Participants were randomly assigned to either an experimental group (H group) or a control group (C group) using an Excel-based number generator.

Randomization and allocation concealment. Participants were allocated 1:1 to the hypoxic (H) or control (C) group by simple randomization. The allocation sequence was generated using the RAND function in Microsoft Excel: each enrolled participant was assigned a unique random number, and the resulting list was sorted in ascending order, with the first half assigned to the H group and the second half to the C group. The randomization list was prepared by an independent researcher who was not involved in participant recruitment, data collection, or outcome assessment. Owing to the nature of the intervention, blinding of the participants and the supervising trainers was not feasible; however, laboratory analyses of blood samples were performed by personnel blinded to group.

The inclusion criteria for both groups were: (1) clinically confirmed hypercholesterolemia, defined as total cholesterol ≥ 5.0 mmol/L (190 mg/dL) and/or LDL-C ≥ 3.0 mmol/L (115 mg/dL) in accordance with the ESC/EAS guidelines for the management of dyslipidaemias; (2) overweight or obesity, defined as a body mass index (BMI) ≥ 25.0 kg/m^2^ in accordance with the WHO classification; (3) age 20–40 years; (4) absence of chronic diseases (i.e., any diagnosed cardiovascular, metabolic/endocrine, respiratory, renal, or hepatic disease, or any condition requiring chronic pharmacotherapy); (5) resting systolic blood pressure < 160 mmHg and diastolic blood pressure < 100 mmHg. The exclusion criteria were: (1) use of illicit drugs, alcohol consumption, or smoking; (2) stage 2 or higher hypertension; (3) premature termination of the exercise test or training program. The baseline characteristics of the participants are presented in Table 4.

All participants underwent a comprehensive medical examination to confirm the absence of contraindications to exercise under hypoxic conditions. Written informed consent was obtained from all participants prior to enrollment, in accordance with ethical guidelines. The study adhered to the principles of the Declaration of Helsinki and was approved by the Bioethics Committee at the University of Zielona Góra, Poland (Resolution No. 6/2023).

### 4.2. Study Design

The experimental protocol consisted of two measurement series: baseline testing (S1) and post-intervention testing (S2). The required sample size was determined using an a priori power analysis performed with G*Power 3.1 software. Assuming an acceptable statistical power of 1 − *β* = 0.80, a significance level of α = 0.05, and an effect size of 0.30, the analysis indicated that a total of *n* = 20 participants would be required to test each research hypothesis. To minimize hormonal variability and its potential impact on lipid profile markers, only male participants were recruited, thereby ruling out any confounding influence of the menstrual cycle on lipid-related variables [49].

Between S1 and S2, over a two-week period, group H underwent passive exposure to normobaric hypoxia (FiO_2_ = 14.4%, 3000 m) at rest and during sleep for 8 h per day in a hypoxic chamber (AirZone 40, Air Sport, Międzyzdroje, Poland). During the stay under hypoxic conditions, blood oxygen saturation (SpO_2_) was continuously recorded to monitor individual responses. In addition, group H performed the IHT protocol (three sessions per week) under hypoxic conditions (FiO_2_ = 14.4%, 3000 m). Group C remained under normoxic conditions at all times, both at rest and during training.

Before the start of the project and taking into account the baseline altitude of the study location (150 m above sea level), all sensors of the hypoxic system were calibrated. The system was equipped with two independent oxygen sensors monitoring O_2_ concentration in each room (gym and hotel rooms) and automatically shut down if a discrepancy greater than 0.4% (corresponding to an altitude difference of ~250 m) was detected. After reaching the target simulated altitude (FiO_2_), the hypoxic system continuously adjusted the gas mixture to maintain stable O_2_ and CO_2_ levels throughout testing. Environmental stability during training sessions was further ensured by the large size of the hypoxic gym (75 m^2^) and continuous monitoring of room conditions. During all training sessions, atmospheric conditions in the hypoxic room were maintained at a constant temperature (19–20 °C) and humidity (50–60%).

Both groups received an identical calorie-restricted diet, designed and supervised by a certified dietitian. All training sessions were supervised and conducted by a personal trainer. Adherence to both the exercise and dietary protocols was monitored throughout the two-week intervention period.

### 4.3. Testing Protocol

In both testing series (S1 and S2), anthropometric and body composition measurements were performed in the morning (8:00–8:30 a.m.) following an overnight fast. Body height was determined with an anthropometer (precision: 0.5 cm), while body composition was assessed by bioelectrical impedance analysis (InBody 220, Biospace, Seoul, South Korea). To standardize conditions across measurements, all participants were required to: (1) maintain an 8 h fast, (2) drink at least 2 L of water the preceding day, (3) refrain from any physical effort for a minimum of 8 h, (4) abstain from caffeine and alcohol for at least 12 h, and (5) avoid diuretics for 24 h prior to testing. Additionally, participants were asked to void their bladder immediately before the assessment [50]. Outcome variables derived from the impedance analysis included fat mass (FM), percentage body fat (%FAT), and fat-free mass (FFM).

Resting metabolic rate (RMR) was subsequently determined by indirect calorimetry using a face mask system (Cortex Metalyser 3B, Leipzig, Germany), previously validated for this purpose [51]. Prior to each measurement, the metabolic analyzer was calibrated using ambient air, a reference gas mixture (5% CO_2_, 15% O_2_), and a 3 L calibration syringe (Hans Rudolph, Shawnee, KS, USA). Participants arrived having fasted for 12 h and having avoided any physical exertion. Measurements were performed with participants lying supine and awake in a thermally (22–23 °C) and humidity-controlled, quiet environment. Gas exchange data were collected over 15 min, with the initial 5 min excluded to allow for stabilization. Only recordings in which a minimum of 5 min of metabolic steady state was achieved—defined as VO_2_ and VCO_2_ fluctuations within ±10%—were retained. Where this criterion was not met, the assessment was rescheduled for the following day, with gas volume variability required to stay within 10% for at least 5 continuous minutes [52].

Venous blood collection took place between 8:30 and 9:00 a.m., directly following the RMR measurement. A 10 mL sample was drawn from the antecubital vein and analyzed for total cholesterol (TC), high-density lipoprotein cholesterol (HDL-C), and triglycerides (TG) using an automated analyzer (Cobas 6000/c501, Roche, Mannheim, Germany). Low-density lipoprotein cholesterol (LDL-C) was derived indirectly via the Friedewald equation: LDL-C = TC − (TG/5 + HDL-C) [53]. One H-group participant with baseline TG > 400 mg/dL (Friedewald validity threshold) was excluded from the LDL-C and CRI-II analyses (*n* = 9 in H for these two variables; *n* = 10 otherwise). Three cardiovascular risk indices were also computed as follows [54,55]:

Atherogenic index of plasma (AIP) = log_10_ (TG/HDL-C);

Castelli’s risk index I (CRI-I) = TC/HDL-C;

Castelli’s risk index II (CRI-II) = LDL-C/HDL-C.

Following blood collection, participants consumed a standardized small meal (350 kcal; 15 g protein, 12.3 g fat, 44.2 g carbohydrates). After a two-hour recovery period, an incremental exercise test was conducted on a cycle ergometer (Excalibur Sport, Lode BV, Groningen, The Netherlands), with workload individually calibrated for each participant. The test began at 50 W and was progressively increased by 25 W every 3 min until the participant reached volitional exhaustion. In the final 15 s of each stage, capillary blood was collected from the fingertip to quantify lactate concentration (LA; LABTREND, BST Bio Sensor Technology GmbH, Berlin, Germany). The resulting lactate kinetics were used to identify the individual lactate threshold by applying the Dmax method [56], which subsequently served as the basis for prescribing individualized training intensities during the intervention.

### 4.4. Training Program

The exercise protocol applied in the present study was identical to that used in our previous investigation [10], with only two modifications: the intervention was shortened from four weeks to two weeks, and nocturnal hypoxic exposure was added in group H. Both groups trained according to the same structured schedule, with workloads individually calibrated to each participant’s physiological capacity. Group H performed the training under normobaric hypoxia (FiO_2_ = 14.4%, 3000 m), while Group C trained under normoxic conditions. Both groups resided at ~100 m above sea level throughout the study. Sessions were held three days per week—Monday, Wednesday, and Friday—and each comprised a 5 min warm-up, an 80 min main training block, and a 5 min cool-down. All intensity zones were prescribed relative to the heart rate corresponding to the individual lactate threshold (HRLT). Specifically, the warm-up was conducted at 70–80% HRLT, whereas the main block required effort at 90–100% HRLT. Within the main block, participants completed four cycling bouts of 10 min each, separated by 10 min active recovery intervals during which exercises for the shoulder girdle, core, and lumbar musculature were performed to sustain overall session intensity. The cool-down consisted of 5 min of low-intensity cycling at 65–70% HRLT. Heart rate was continuously recorded throughout all sessions using a wrist-based monitor (Polar Pacer, Polar Electro Oy, Kempele, Finland). In group H, arterial oxygen saturation (SpO_2_) was additionally tracked via a dedicated pulse oximeter (WristOx2 3150, Nonin Medical Inc., Plymouth, MN, USA). All sessions were delivered by a certified personal trainer under the ongoing supervision of the research team.

### 4.5. Diets During the Experiment

The nutritional protocol applied in the present study was identical to that used in our previous investigation [10]. All participants adhered to the same training schedule and nutritional plan throughout the study. Dietary intake consisted of a controlled mixed diet characterized by the following macronutrient distribution: 45.2 ± 3.5% carbohydrates, 23.8 ± 2.7% fat, and 30.6 ± 2.7% protein (Table 5). Total daily energy intake was set at approximately 2517.4 ± 43.4 kcal and individually adjusted for each participant on the basis of resting metabolic rate values obtained via indirect calorimetry (Table 1). Daily energy was distributed across five eating occasions: breakfast, mid-morning snack, lunch, afternoon snack, and dinner. All meals were prepared, weighed, and portioned on an individual basis to ensure precise dietary control. To facilitate full supervision of food consumption, participants resided at the study facility for the entire duration of the intervention, eliminating the possibility of uncontrolled dietary intake outside the prescribed protocol. Fluid intake was standardized according to activity level, with a minimum of 2.5 L of water required on rest days and at least 3.5 L on training days [57]. For the duration of the study, participants were instructed to abstain from alcohol, nutritional supplements, and any pharmacological agents. Dietary compliance was monitored continuously throughout the two-week intervention period. Additionally, daytime physical activity was continuously recorded using HR watches (Pacer, Polar Electro Oy, Kempele, Finland), and participants adhered to the prescribed training requirements, with intensive exercise limited to the training sessions.

### 4.6. Statistical Analysis

All data were analyzed using Statistica v.13 (StatSoft, Tulsa, OK, USA). Results are presented as arithmetic means ($\bar{x}$) ± standard deviations (SD). The normality of distributions was verified using the Shapiro–Wilk test, and homogeneity of variance was assessed with Levene’s test. When assumptions for parametric testing were met, a two-way repeated-measures ANOVA was applied. If significant effects were observed, Tukey’s post hoc analyses were conducted. To compare between-group differences in delta values of the studied variables, independent-samples *t*-tests were used. When normality assumptions were violated, the Wilcoxon test was applied as a non-parametric alternative for paired data (before vs. after intervention within a group), and the Mann–Whitney U test was used to compare independent samples (between groups H and C). Effect sizes were calculated using Hedges’ *g*. Ninety-five percent confidence intervals (95% CI) were calculated for all effect size estimates. Effect sizes were interpreted as trivial (<0.20), small (0.20–0.49), moderate (0.50–0.79), and large (≥0.80). Pearson’s correlation analysis was performed to assess relationships between selected variables. For all analyses, statistical significance was set at *p* < 0.05.

## 5. Conclusions

The present study demonstrates that a two-week intervention combining IHT with passive nocturnal exposure (8 h/day, FiO_2_ = 14.4%, ~3000 m) and a controlled hypocaloric diet produces clinically meaningful improvements in lipid profile and body composition in physically inactive men with obesity and secondary hypercholesterolemia. Significant reductions in TC, LDL-C, non-HDL-C, and AIP were observed exclusively in the H group, alongside greater reductions in body mass and fat mass compared with the normoxic control group. The absence of significant lipid or body composition changes in the C group—despite adherence to an identical training and dietary protocol—indicates that hypoxia was the key differentiating factor driving these metabolic adaptations.

The marked improvement in AIP—widely regarded as one of the most sensitive composite markers of atherogenic risk—together with the significant reduction in CRI-I and the strong downward trend in CRI-II, suggests that the combined hypoxic protocol may enhance favorable lipoprotein remodeling and contribute to a reduction in estimated cardiovascular risk. The significant correlations between body mass loss and improvements in TC and LDL-C, and between fat mass loss and non-HDL-C reduction, indicate that anthropometric changes are key mediators of the lipid response and that the hypoxic stimulus amplifies this relationship. Notably, fat-free mass and resting metabolic rate were preserved in both groups, confirming that the dietary strategy protected muscle mass—an important consideration in obesity treatment.

Compared with our previous four-week IHT protocol [10], the present two-week protocol supplemented with nocturnal hypoxic exposure produced comparable reductions in TC, LDL-C, and non-HDL-C, and a nearly twofold greater improvement in AIP in half the intervention time. This finding supports the concept that the total daily dose of hypoxic exposure, rather than hypoxic training alone, may constitute the principal determinant of lipid-related adaptation, and suggests that exposure duration warrants consideration as a primary dosing variable in the design of future hypoxic interventions.

From a clinical perspective, the proposed protocol represents a time-efficient, non-pharmacological strategy capable of producing meaningful improvements in cardiovascular risk markers in physically inactive men with obesity-related hypercholesterolemia, within a timeframe compatible with short-term hospital or rehabilitation settings. Future studies with larger samples, longer follow-up, and direct molecular assessment of HIF-1α and PGC-1α activation are needed to confirm and extend these findings.

## Figures and Tables

**Table 1:** Body mass and composition of participants before (S1) and after training (S2) in the hypoxic training (H) and control (C) groups.

Variables	H Group		C Group	
	Before (S1)	After (S2)	Before (S1)	After (S2)
BM (kg)	96.5 ± 7.7	92.6 ± 7.8 ***	89.8 ± 8.8	88.2 ± 8.1 ***
%FAT	29.2 ± 5.6	26.6 ± 5.7 **	28.5 ± 7.1	27.9 ± 6.9
FM (kg)	28.4 ± 5.9	25.3 ± 5.5 ***	26.0 ± 7.9	25.0 ± 7.5
FFM (kg)	68.2 ± 8.4	67.6 ± 8.0	63.8 ± 4.9	63.2 ± 4.3
RMR (kcal/day)	2399.1 ± 168.0	2382.8 ± 214.0	2403.0 ± 133.5	2308.1 ± 213.7

Abbreviations: BM—body mass; %FAT—fat content; FM—fat mass; FFM—fat-free mass; RMR—resting metabolic rate; ** *p* < 0.01, *** *p* < 0.001 before vs. after training.

**Table 2:** Lipid profile of participants before (S1) and after training (S2) in the hypoxic training (H) and control (C) groups.

Variables	H Group		C Group	
	Before (S1)	After (S2)	Before (S1)	After (S2)
TC (mg/dL)	212.4 ± 22.5	174.4 ± 23.2 ***	225.8 ± 30.8	207.4 ± 17.2
HDL-C (mg/dL)	40.9 ± 12.9	38.7 ± 6.0	50.5 ± 12.3	47.6 ± 14.2
LDL-C (mg/dL)	148.6 ± 21.2	111.6 ± 26.7 **	147.1 ± 23.0	135.3 ± 14.2
non-HDL-C (mg/dL)	171.2 ± 20.7	134.9 ± 19.4 ***	175.0 ± 35.6	158.9 ± 15.1
TG (mg/dL)	191.6 ± 179.5	124.3 ± 137.0	155.7 ± 117.8	134.4 ± 66.2

Abbreviations: TC—total cholesterol; HDL-C—high-density lipoprotein cholesterol; LDL-C—low-density lipoprotein cholesterol; non-HDL-C non-high-density lipoprotein cholesterol; TG—triglycerides; ** *p* < 0.01, *** *p* < 0.001 before vs. after training.

**Table 3:** Atherogenic indices in participants before (S1) and after intervention (S2) in hypoxia (H) and control groups (C).

Variables	H Group		C Group	
	Before (S1)	After (S2)	Before (S1)	After (S2)
AIP	0.59 ± 0.33	0.34 ± 0.39 **	0.40 ± 0.37	0.42 ± 0.28
CRI-I	5.63 ± 1.59	4.54 ± 0.69 *	4.74 ± 1.51	4.66 ± 1.22
CRI-II	3.91 ± 1.51	3.11 ± 0.63	3.11 ± 1.08	3.08 ± 0.97

AIP—atherogenic index of plasma: log_10_(TG/HDL-C); CRI-I—Castelli’s risk index I: TC/HDL-C; CRI-II—Castelli’s risk index II: LDL-C/HDL-C; * *p* < 0.05, ** *p* < 0.01 before vs. after training.

**Table 4:** Baseline characteristics of the participants.

Variables	H Group	C Group
Age (years)	36.6 ± 4.6	35.8 ± 4.9
Body height (cm)	175.7 ± 4.3	172.9 ± 3.6
Body mass (kg)	96.5 ± 7.7	89.8 ± 8.8
Body fat (%)	29.2 ± 5.6	28.5 ± 7.1
Systolic blood pressure (mmHg)	138.9 ± 7.8	142.2 ± 16.7
Diastolic blood pressure (mmHg)	87.9 ± 9.47	94.0 ± 11.2

**Table 5:** Nutritional composition during the study.

Protein (g)	Fat (g)	Carbohydrates (g)	Caloric Intake (kcal)
192.0 ± 15.0	66.5 ± 7.8	285.1 ± 22.0	2517.4 ± 43.4

## Data Availability

The original contributions presented in this study are included in the article. Further inquiries can be directed to the corresponding author.

## Abbreviations

The following abbreviations are used in this manuscript:

%FAT	Body fat percentage
AIP	Atherogenic index of plasma
BH	Body height
BM	Body mass
BMI	Body mass index
CRI-I	Castelli Risk Index I
CRI-II	Castelli Risk Index II
CVD	Cardiovascular disease
FFM	Fat-free mass
FM	Fat mass
HDL-C	High-density lipoprotein cholesterol
HIF-1	Hypoxia-inducible factor
HRLT	Heart rate at lactate threshold
IHT	Intermittent hypoxic training
LDL-C	Low-density lipoprotein cholesterol
PGC-1α	Peroxisome proliferator-activated receptor-gamma coactivator 1α
RMR	Resting metabolic rate
SD	Standard deviations
TC	Total cholesterol
TG	Triglycerides

## References

[1] Cannon C.P. (2007). Cardiovascular disease and modifiable cardiometabolic risk factors. Clin. Cornerstone.

[2] Pirillo A., Casula M., Olmastroni E., Norata G.D., Catapano A.L. (2021). Global epidemiology of dyslipidaemias. Nat. Rev. Cardiol..

[3] Poobalan A., Aucott L., Smith W.C., Avenell A., Jung R., Broom J., Grant A.M. (2004). Effects of weight loss in overweight/obese individuals and long-term lipid outcomes—A systematic review. Obes. Rev..

[4] Millet G.P., Debevec T., Brocherie F., Malatesta D., Girard O. (2016). Therapeutic Use of Exercising in Hypoxia: Promises and Limitations. Front. Physiol..

[5] Semenza G.L. (2012). Hypoxia-inducible factors in physiology and medicine. Cell.

[6] Zoll J., Ponsot E., Dufour S., Doutreleau S., Ventura-Clapier R., Vogt M., Hoppeler H., Richard R., Flück M. (2006). Exercise training in normobaric hypoxia in endurance runners. III. Muscular adjustments of selected gene transcripts. J. Appl. Physiol..

[7] Luo J., Yang H., Song B.L. (2020). Mechanisms and regulation of cholesterol homeostasis. Nat. Rev. Mol. Cell Biol..

[8] Adiels M., Taskinen M.R., Borén J. (2008). Fatty liver, insulin resistance, and dyslipidemia. Curr. Diabetes Rep..

[9] Niemaszyk A., Płoszczyca K., Czuba M. (2025). The use of intermittent hypoxic training in rehabilitation, prevention, and treatment of non-communicable diseases (NCDs): A narrative review. Biomed. Hum. Kinet..

[10] Jędrzejewski E., Czuba M., Niemaszyk A., Płoszczyca K., Kaczmarczyk K., Langfort J., Gajda R. (2025). Hypoxic Training with Calorie Restriction Improves Lipid Profile and Body Composition in Men with Obesity-Related Hypercholesterolemia: A Controlled Intervention Study. Int. J. Mol. Sci..

[11] Ghaith A., Chacaroun S., Borowik A., Chatel L., Doutreleau S., Wuyam B., Tamisier R., Pépin J.L., Flore P., Verges S. (2022). Hypoxic high-intensity interval training in individuals with overweight and obesity. Am. J. Physiol. Regul. Integr. Comp. Physiol..

[12] Fernández Menéndez A., Saudan G., Sperisen L., Hans D., Saubade M., Millet G.P., Malatesta D. (2018). Effects of Short-Term Normobaric Hypoxic Walking Training on Energetics and Mechanics of Gait in Adults with Obesity. Obesity.

[13] Klug L., Mähler A., Rakova N., Mai K., Schulz-Menger J., Rahn G., Busjahn A., Jordan J., Boschmann M., Luft F.C. (2018). Normobaric hypoxic conditioning in men with metabolic syndrome. Physiol. Rep..

[14] Thackray A.E., Stensel D.J. (2023). The impact of acute exercise on appetite control: Current insights and future perspectives. Appetite.

[15] Płoszczyca K., Czuba M., Langfort J., Baranowski M. (2021). Exposure to Normobaric Hypoxia Combined with a Mixed Diet Contributes to Improvement in Lipid Profile in Trained Cyclists. Nutrients.

[16] Lizamore C.A., Hamlin M.J. (2017). The Use of Simulated Altitude Techniques for Beneficial Cardiovascular Health Outcomes in Nonathletic, Sedentary, and Clinical Populations: A Literature Review. High Alt. Med. Biol..

[17] He Z., Qiang L., Liu Y., Gao W., Feng T., Li Y., Yan B., Girard O. (2023). Effect of Hypoxia Conditioning on Body Composition in Middle-Aged and Older Adults: A Systematic Review and Meta-Analysis. Sports Med. Open.

[18] Ramos-Campo D.J., Girard O., Pérez A., Rubio-Arias J. (2019). Additive stress of normobaric hypoxic conditioning to improve body mass loss and cardiometabolic markers in individuals with overweight or obesity: A systematic review and meta-analysis. Physiol. Behav..

[19] Burtscher M. (2013). Effects of living at higher altitudes on mortality: A narrative review. Aging Dis..

[20] Dominguez Coello S., Cabrera De León A., Bosa Ojeda F., Pérez Méndez L.I., Díaz González L., Aguirre-Jaime A.J. (2020). High density lipoprotein cholesterol increases with living altitude. Int. J. Epidemiol..

[21] Mohanna S., Baracco R., Seclen S. (2006). Lipid profile, waist circumference, and body mass index in a high altitude population. High Alt. Med. Biol..

[22] Sharma S. (1990). Clinical, biochemical, electrocardiographic and noninvasive hemodynamic assessment of cardiovascular status in natives at high to extreme altitudes (3000m-5500m) of the Himalayan region. Indian Heart J..

[23] Férézou J., Richalet J.P., Coste T., Rathat C. (1988). Changes in plasma lipids and lipoprotein cholesterol during a high altitude mountaineering expedition (4800 m). Eur. J. Appl. Physiol. Occup. Physiol..

[24] Verratti V., Falone S., Doria C., Pietrangelo T., Di Giulio C. (2015). Kilimanjaro Abruzzo expedition: Effects of high-altitude trekking on anthropometric, cardiovascular and blood biochemical parameters. Sport Sci. Health.

[25] Gutwenger I., Hofer G., Gutwenger A.K., Sandri M., Wiedermann C.J. (2015). Pilot study on the effects of a 2-week hiking vacation at moderate versus low altitude on plasma parameters of carbohydrate and lipid metabolism in patients with metabolic syndrome. BMC Res. Notes.

[26] Minvaleev R.S. (2011). Comparison of the rates of changes in the lipid spectrum of human blood serum at moderate altitudes. Hum. Physiol..

[27] Greie S., Humpeler E., Gunga H.C., Koralewski E., Klingler A., Mittermayr M., Fries D., Lechleitner M., Hoertnagl H., Hoffmann G. (2006). Improvement of metabolic syndrome markers through altitude specific hiking vacations. J. Endocrinol. Investig..

[28] Voss J.D., Masuoka P., Webber B.J., Scher A.I., Atkinson R.L. (2013). Association of elevation, urbanization and ambient temperature with obesity prevalence in the United States. Int. J. Obes..

[29] Woolcott O.O., Gutierrez C., Castillo O.A., Elashoff R.M., Stefanovski D., Bergman R.N. (2016). Inverse association between altitude and obesity: A prevalence study among Andean and low-altitude adult individuals of Peru. Obesity.

[30] Sherpa L.Y., Deji, Stigum H., Chongsuvivatwong V., Thelle D.S., Bjertness E. (2010). Obesity in Tibetans aged 30–70 living at different altitudes under the north and south faces of Mt. Everest. Int. J. Environ. Res. Public Health.

[31] Gao H., Xu J., Zhang L., Lu Y., Gao B., Feng L. (2020). Effects of Living High-Training Low and High on Body Composition and Metabolic Risk Markers in Overweight and Obese Females. BioMed Res. Int..

[32] Thompson P.D., Jeffery R.W., Wing R.R., Wood P.D. (1979). Unexpected decrease in plasma high density lipoprotein cholesterol with weight loss. Am. J. Clin. Nutr..

[33] Brinton E.A., Eisenberg S., Breslow J.L. (1990). A low-fat diet decreases high density lipoprotein (HDL) cholesterol levels by decreasing HDL apolipoprotein transport rates. J. Clin. Investig..

[34] Andraschko L.M., Gazi G., Leucuta D.C., Popa S.L., Chis B.A., Ismaiel A. (2025). Atherogenic Index of Plasma in Metabolic Syndrome-A Systematic Review and Meta-Analysis. Medicina.

[35] Castelli W.P. (1988). Cholesterol and lipids in the risk of coronary artery disease—The Framingham Heart Study. Can. J. Cardiol..

[36] Castelli W.P., Anderson K., Wilson P.W., Levy D. (1992). Lipids and risk of coronary heart disease. The Framingham Study. Ann. Epidemiol..

[37] Olamoyegun M.A., Oluyombo R., Asaolu S.O. (2016). Evaluation of dyslipidemia, lipid ratios, and atherogenic index as cardiovascular risk factors among semi-urban dwellers in Nigeria. Ann. Afr. Med..

[38] Baigent C., Blackwell L., Emberson J., Holland L.E., Reith C., Bhala N., Peto R., Barnes E.H., Keech A., Cholesterol Treatment Trialists’ (CTT) Collaboration (2010). Efficacy and safety of more intensive lowering of LDL cholesterol: A meta-analysis of data from 170,000 participants in 26 randomised trials. Lancet.

[39] Wang B., Liu J., Chen S., Ying M., Chen G., Liu L., Lun Z., Li H., Huang H., Li Q. (2021). Malnutrition affects cholesterol paradox in coronary artery disease: A 41,229 Chinese cohort study. Lipids Health Dis..

[40] Sato R., Matsuzawa Y., Yoshii T., Akiyama E., Konishi M., Nakahashi H., Minamimoto Y., Kimura Y., Okada K., Maejima N. (2024). Impact of Low-Density Lipoprotein Cholesterol Levels at Acute Coronary Syndrome Admission on Long-Term Clinical Outcomes. J. Atheroscler. Thromb..

[41] Chen M., Zhang L., Liu Q., Gu Q., Yu S., Lu G. (2025). Non-high density lipoprotein cholesterol/high density lipoprotein cholesterol is L-shaped associated with all-cause mortality and U-shaped with cardiovascular mortality in hypertensive patients. Front. Endocrinol..

[42] Straniero S., Rosqvist F., Edholm D., Ahlström H., Kullberg J., Sundbom M., Risérus U., Rudling M. (2017). Acute caloric restriction counteracts hepatic bile acid and cholesterol deficiency in morbid obesity. J. Intern. Med..

[43] Seip R.L., Semenkovich C.F. (1998). Skeletal muscle lipoprotein lipase: Molecular regulation and physiological effects in relation to exercise. Exerc. Sport Sci. Rev..

[44] Hawley J.A., Hargreaves M., Joyner M.J., Zierath J.R. (2014). Integrative biology of exercise. Cell.

[45] Klop B., Elte J.W., Cabezas M.C. (2013). Dyslipidemia in obesity: Mechanisms and potential targets. Nutrients.

[46] Mungai P.T., Waypa G.B., Jairaman A., Prakriya M., Dokic D., Ball M.K., Schumacker P.T. (2011). Hypoxia triggers AMPK activation through reactive oxygen species-mediated activation of calcium release-activated calcium channels. Mol. Cell Biol..

[47] Ponsot E., Dufour S.P., Zoll J., Doutreleau S., N’Guessan B., Geny B., Hoppeler H., Lampert E., Mettauer B., Ventura-Clapier R. (2006). Exercise training in normobaric hypoxia in endurance runners. II. Improvement of mitochondrial properties in skeletal muscle. J. Appl. Physiol..

[48] Lecoultre V., Peterson C.M., Covington J.D., Ebenezer P.J., Frost E.A., Schwarz J.-M., Ravussin E. (2013). Ten nights of moderate hypoxia improves insulin sensitivity in obese humans. Diabetes Care.

[49] Bergström H., Ekström L., Warnqvist A., Bergman P., Björkhem-Bergman L. (2021). Variations in biomarkers of dyslipidemia and dysbiosis during the menstrual cycle: A pilot study in healthy volunteers. BMC Women’s Health.

[50] Vogler A.J., Rice A.J., Gore C.J. (2010). Validity and reliability of the cortex MetaMax3B portable metabolic system. J. Sports Sci..

[51] Schiavo L., Scalera G., Pilone V., De Sena G., Iannelli A., Barbarisi A. (2017). Fat mass, fat-free mass, and resting metabolic rate in weight-stable sleeve gastrectomy patients compared with weight-stable nonoperated patients. Surg. Obes. Relat. Dis..

[52] Almajwal A.M., Abulmeaty M.J. (2019). New predictive equations for resting energy expenditure in normal to overweight and obese population. Int. J. Endocrinol..

[53] Friedewald W.T., Levy R.I., Fredrickson D.S. (1972). Estimation of the concentration of low-density lipoprotein cholesterol in plasma, without use of the preparative ultracentrifuge. Clin. Chem..

[54] Castelli W.P., Abbott R.D., McNamara P.M. (1983). Summary estimates of cholesterol used to predict coronary heart disease. Circulation.

[55] Dobiášová M., Frohlich J. (2001). The plasma parameter log (TG/HDL-C) as an atherogenic index: Correlation with lipoprotein particle size and esterification rate in apoB-lipoprotein-depleted plasma (FERHDL). Clin. Biochem..

[56] Cheng B., Kuipers H., Snyder A.C., Keizer H.A., Jeukendrup A., Hesselink M.A. (1992). New Approach for the determination of ventilatory and lactate thresholds. Int. J. Sports Med..

[57] San Mauro M.I., Garicano V.E., Romo Orozco D.A., Mendive Dubourdieu P., Paredes Barato V., Rincón Barrado M., Valente A., Bentancor F., Morales Hurtado A.D., Garagarza C. (2017). Hydration Status: Influence of Exercise and Diet Quality. Am. J. Lifestyle Med..

